# An Elegant Biosensor Molecular Beacon Probe: Challenges and Recent Solutions

**DOI:** 10.6064/2012/928783

**Published:** 2012-12-13

**Authors:** Dmitry M. Kolpashchikov

**Affiliations:** Chemistry Department, University of Central Florida, 4000 Central Florida Boulevard, Orlando, FL 32816-2366, USA

## Abstract

Molecular beacon (MB) probes are fluorophore- and quencher-labeled short synthetic DNAs folded in a stem-loop shape. Since the first report by Tyagi and Kramer, it has become a widely accepted tool for nucleic acid analysis and triggered a cascade of related developments in the field of molecular sensing. The unprecedented success of MB probes stems from their ability to detect specific DNA or RNA sequences immediately after hybridization with no need to wash out the unbound probe (instantaneous format). Importantly, the hairpin structure of the probe is responsible for both the low fluorescent background and improved selectivity. Furthermore, the signal is generated in a reversible manner; thus, if the analyte is removed, the signal is reduced to the background. This paper highlights the advantages of MB probes and discusses the approaches that address the challenges in MB probe design. Variations of MB-based assays tackle the problem of stem invasion, improve SNP genotyping and signal-to-noise ratio, as well as address the challenges of detecting folded RNA and DNA.

## 1. Introduction: An Elegant Unimolecular Biosensor

**Figure 1:**
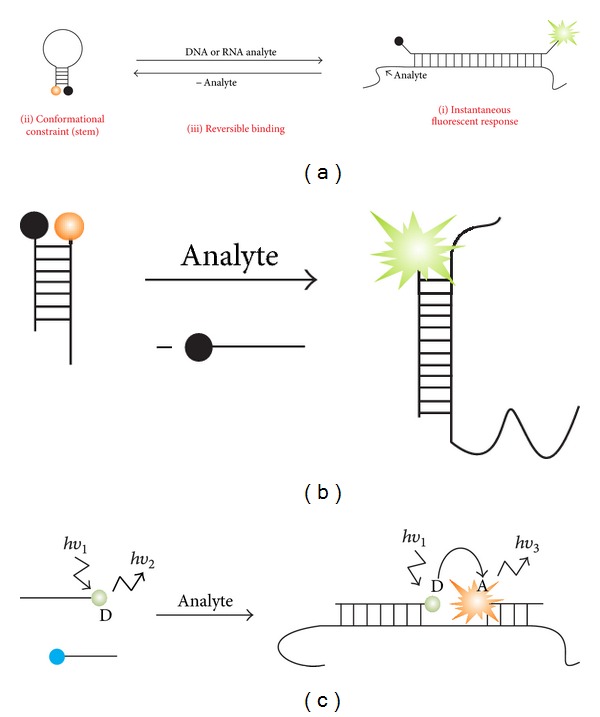
Instantaneous hybridization probes. (a) Classical design of molecular beacon (MB) probe [1–3]. Some important features of the probe are (i) the ability to produce instantaneous fluorescent signal; (ii) conformational constraint in the form of a stem loop in the target-unbound state (left); (iii) reversibility of hybridization. (b) Strand displacement probes [7–10]. The quencher-conjugated strand is displaced from the complex with the fluorophore-conjugated DNA by the analyte. (c) Adjacent probes [11–15]. Hybridization of two fluorophore-conjugated DNA probes to the adjacent positions of an analyte results in Förster resonance energy transfer (FRET). The hybridization efficiency is assessed as a difference in donor and acceptor fluorescence.

In its classical design [1–3], a molecular beacon (MB) probe is a stem-loop-folded oligodeoxyribonucleotide with fluorophore and quencher dyes conjugated to the opposite ends of the hairpin (Figure 1(a)). In the absence of a complementary nucleic acid target (analyte), the fluorescence of the fluorophore is quenched by the closely located quencher dye. Formation of the probe-analyte duplex separates the fluorophore from the quencher, thus brightening the MB's fluorescence. The emitted light can be quantified directly in the sample. The behavior of this molecule can be considered as an elementary molecular device that switches between the two conformations in an analyte-dependent manner. Therefore, MB probes have forestalled the rise of DNA nanomotors and nanorobots, a field that has recently received a substantial attention [4–6].

The most important features of MB probe include (i) s generation of fluorescent signal that can be registered immediately after hybridization event; (ii) conformational constraint in the form of a stem loop; (iii) reversible binding to the analyte (Figure 1(a)) as detailed below. 

**Figure 2:**
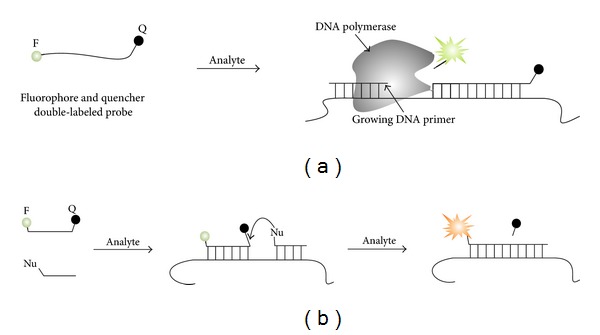
Examples of fluorescent reporters. (a) TaqMan probe. Fluorophore- and quencher-labeled stem-free oligonucleotide hybridizes to a single-stranded DNA amplicon and becomes cleaved by the 5′–>3′ exonuclease activity of DNA polymerase during elongation step of rtPCR [16–18]. (b) Chemical autoligation assay [18–20]. An oligonucleotide with a fluorophore and a quencher hybridizes to the analyte in proximity to the second oligonucleotide probe equipped with a nucleophilic reactive group. Chemical reaction of the two oligonucleotide probes results in both ligation of the oligonucleotides and detachment of the quencher. The unquenched fluorophore produces bright fluorescence.

(i) The probe produces a signal that enables the detection of the target immediately in homogeneous solution without the need for separation of the probe-analyte hybrid from the excess amount of the unbound probe. This property of a probe sometimes is referred to as “real time.” However, this term is traditionally associated with real-time PCR (rtPCR) that also uses SYBR Green, and TaqMan assay, which significantly differ from MB probe and other immediate or instant mix-and-read type of assays. MB probes have broader spectrum of applications and greater significance than just rtPCR. Therefore, here we use the terms “instantaneous format” and “instantaneous probes” to define the property of MB probes to be used without washing steps. The significance of MB probe can be better understood in the context of preceding instantaneous probes such as strand displacement probes (Figure 1(b)) and adjacent hybridization probes (Figure 1(c)). Strand displacement probe with a fluorophore on one strand and a quencher on the other strand was introduced by Morrison et al. [7, 8] and used for the fluorescent detection of hybridization events in a number of studies [9, 10, 21–26]. The approach requires synthesis and purification of two labeled oligonucleotides followed by titration of the fluorophore strand with the quencher oligonucleotide. Overall, these procedures are more effort intensive in comparison with the synthesis and purification of a single MB probe. Moreover, once separated by heat or by binding to nonspecific biopolymers, the two strands have lower chance to quantitatively reassociate, which may lead to elevated background in complex systems. Adjacent hybridization probes [11–14] use Förster resonance energy transfer (FRET) between two dye-conjugated oligonucleotides hybridized to the adjacent positions of the analyte. They have been extensively studied since the 80s and employed for LightCycler rtPCR technology [15]. FRET-based probes, however, create high background noise due to the overlap between the emission spectra of the donor and acceptor fluorophores, as well as due to partial excitation of the acceptor at the excitation wavelength of the donor [11, 14]. In addition, FRET efficiency is very sensitive to the arrangement of the two dyes, thus requiring optimization of the hybridization sites with the most efficient FRET occurring if the two oligonucleotides hybridize at the distance of 1–5 nucleotides [27]. Overall, the unimolecular nature and FRET independence of operation contribute to the great success of MB probes. 

(ii) The complementary ends of MB probe determine its stem-loop shape, which is important both for the low background fluorescence in the target-unbound form as well as for the improved selectivity in comparison with linear oligonucleotide probes. This secondary structure brings the fluorophore in proximity to the quencher, thus enabling efficient “contact quenching” [28]. This type of quenching occurs only for closely located fluorophore-quencher pairs and does not require overlap of fluorophore emission spectrum with quencher absorption spectrum. In addition, the secondary structure is a form of conformational constraint [29–31] that imparts extraordinary selectivity: the probe would hybridize to the target only if a significant energy gain is offered, thus rejecting mismatched targets. This property of MB probes is used to differentiate analytes with single-nucleotide differences, which is practically important for the analysis of single-nucleotide polymorphisms (SNPs) as well as point mutations. 

(iii) The probe hybridizes to the target in a reversible manner, thus enabling dynamic readout. This property of MB probe is underrecognized, yet important, as it can find a number of interesting applications including spatiotemporal monitoring of changes in intracellular concentration of specific RNAs. Probes that accumulate signal over time and maintain it in the absence of analyte are significantly different and shall be classified as reporters rather than sensors [32]. For example, enzyme-dependent TaqMan probe [33–35] and chemical-ligation probes [16, 36, 37] accumulate fluorescence during their exposure to the target (Figure 2). These and other reporters including “catalytic molecular beacons” [17–20, 38–45] and MB probe-based nuclease-assisted assays [46–53] are beyond the scope of this spotlight paper.

The design and application of MB probes were the subjects of a number of excellent reviews [54–73]. A recent overview of MB probe technology was accomplished by Guo et al. [54] and Li et al. [55]. These reviews, as well as works by Silverman and Kool [56], Ihara and Kitamura [57], and Broude [58], put MB probe in the context of related technologies. The design of MB probes was addressed in reviews by Marras [28], Wang et al., [59], Huang and Martí [60], and Venkatesan et al. [61]. Some reviews focus on particular applications of MB probes. For example, Marras et al. [62], Juskowiak [63], and Buh Gašparič et al. [64] compared MB probe with other rtPCR chemistries. Recent extensive attention is devoted to application of the MB probe for dynamic monitoring of RNA in cells [66–70], which reflects the urgent need for the intracellular fluorescent RNA imaging. A number of reviews describe application of MB probes for the study of proteins [71–73]. Overall, such wide expansion of the probe reflects branching of the field, thus testifying the fruitfulness of MB approach. The purposes of this spotlight paper are (i) highlighting the advantages of MB probe in the context of related technologies; (ii) critical analysis of the challenges in MB probe design; (iii) discussion of the most promising strategies to address the existing challenges; (iv) updating with most recent variants of MB-inspired sensors.

## 2. Challenges

### 2.1. MB Probe Design

MB probe falls under the IUPAC definition for “biosensor”: “a biosensor is a compact analytical device incorporating a biological or biologically derived sensing element, either integrated within or intimately associated with a physicochemical transducer” [84]. Indeed, the loop portion of the DNA probe is a sensing element, while the stem portion together with a fluorophore and a quencher can be considered as a physicochemical transducer. This composition implies the need of optimizing both the stem and the loop portions of MB probe in accordance with their functions to achieve the best possible performance.

The design of MB probe can be assisted using Beacon Designer software [85]. However, in many cases, common considerations are sufficient for the acceptable results. Overall, to favor the formation of the probe-target complex, the melting temperature of the loop portion should be higher than that of the stem. The loop is typically 15–20 nucleotides long and fully complementary to the analyte. The stem should be C/G rich and contain 4–7 base pairs to ensure high stability and acceptable hybridization rates. Longer and more stable stems will reduce hybridization rates but may improve assay selectivity [86]. The melting temperature of the stem should be at least 7°C higher than the assay temperature to ensure efficient fluorescent quenching in the free MB probe [87]. If the assay is SNP specific, the interrogated position should be complementary to a nucleotide close to the middle position of the loop sequence for better allele differentiation. To provide low background of the assay, the quantum yield of the fluorescent dye and the quenching efficiency of the nonfluorescent dye should be taken into account [28]. A variety of combinations of fluorophore/quencher pairs are commercially available [88, 89]. Interestingly, a single dark quencher such as DABCYL (4-((4-(dimethylamino)phenyl)azo)benzoic acid) can quench a great variety of fluorophores, even those that do not have spectral overlap with DABCYL [28, 62]. Fluorophore dye should not be conjugated to guanosine as guanine residue can quench its fluorescence by as much as 40% depending upon the fluorophore [90, 91].

**Figure 3:**
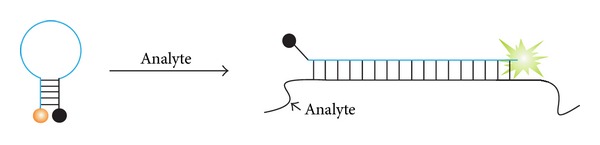
Shared-stem MB probe. A part of stem-forming nucleotides is complementary to the target sequence. MB fragment complementary to the analyte is shown in cyan.

An option for the “shared-stem” probe is available [92]. In this variation, the target-recognition element of the probe includes not only the loop portion of MB probe but also one arm of the stem (Figure 3). This design shifts the equilibrium towards the probe-analyte complex, thus increasing the signal of the probe in the presence of the target. However, such probes display reduced hybridization selectivity [93–97]. In addition, it implies structural restrictions for the choice of the stem sequence, which may not be acceptable for AT-rich analytes. At the same time, partial stem complementarity (1–3 bases) to the target analyte may improve the affinity and increase signal-to-noise (S/N) ratio without compromising the probe's selectivity [98].

The aforementioned design of MB probe may look straightforward and simple. In practice, however, there are significant complications to the degree that it is impossible to design an MB probe for some particular analyte sequences at all.

### 2.2. Interference of Loop or Stem Nucleotides

**Figure 4:**
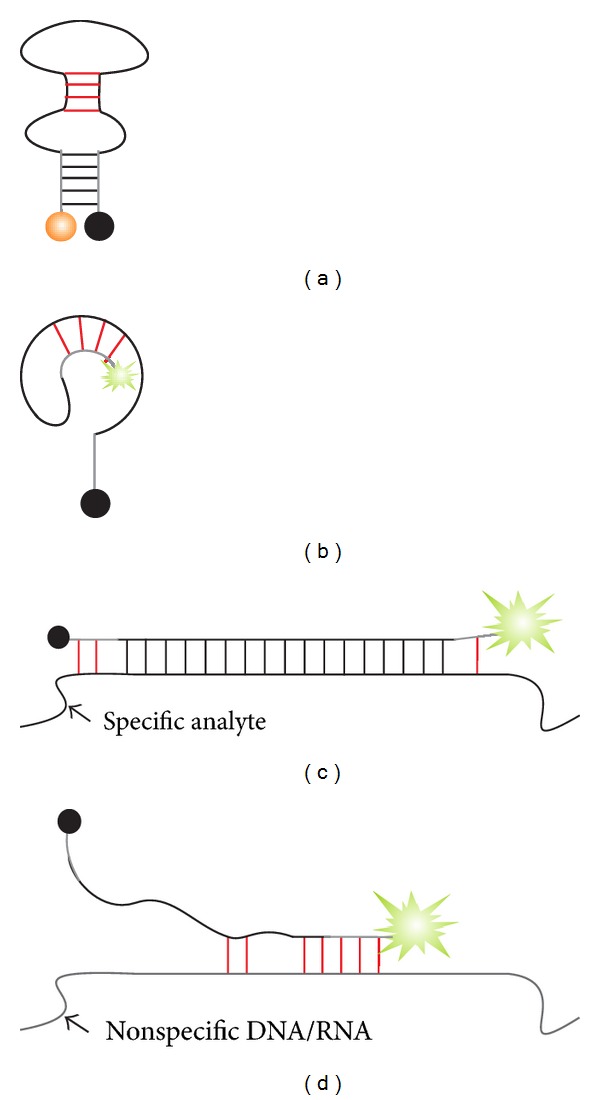
Examples of loop and stem interferences in MB probe. (a) Loop interference. Complementary loop nucleotides hybridize to each other, thus making folded conformation more stable and less accessible by the target analyte. (b) Stem hybridizes to loop thus separating the fluorophore from the quencher resulting in elevated background signal. (c) Stem nucleotides can partially hybridize to the nucleotides of the targeted analyte. (d) Stem and loop nucleotides bind nonspecific nucleic acids present in a sample. Red lines indicate undesired or undesigned base pairs.

The folding and hybridization of MB probe and, therefore, its predicted performance can be affected by the interference of loop or stem sequences (Figure 4). The loop sequence is predetermined by the analyte, which imposes significant limitations in probe's design. For example, if an SNP site is targeted, the loop allows only minor variation of shifting several nucleotides towards the 3′- or 5′-end along the analyte sequence without compromising probe selectivity. On the other hand, the loop sequence can appear to be partially self-complementary (Figure 4(a)). This additional secondary structure element stabilizes the closed form of MB probe and may both slow hybridization kinetics and reduce hybridization efficiency. 

In addition, the stem nucleotides should be chosen to avoid complementarity with the loop. Otherwise, an alternative structure might be formed, in which fluorophore is not efficiently quenched (Figure 4(b)). This imposes a limitation on the choice for stem sequences as well as requires an alteration of stem sequences for each new MB probe. Additional limitation on the choice of stem sequences is applied by the possibility of stem invasion (Figures 4(c) and 4(d)). Stem nucleotides may interact with the analyte nucleotides that flank the target site (red lines in Figure 4(c)). These interactions are almost inevitable considering C/G-rich nature of stems and the possibility of hydrogen bond formation between G and any another base [99]. This type of interactions is hard yet important to predict as they affect both affinity and selectivity of the probe-target interactions.

**Figure 5:**
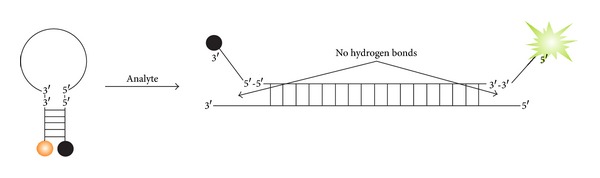
Hairpin inversion MB probes [100]. The terminal stem-forming sequences are connected by the 3′-3′ or 5′-5′ phosphodiester linkages (left). In complex with a target, the orientation of DNA strand in stem-forming fragments is the same as in the analyte, which eliminates a possibility of hybridization.

The aforementioned complications would have been eliminated if stem and loop could be designed and functioned independently. To achieve this, hairpin inversion MB probes were suggested by Browne [100]. In this variation of MB probes, the orientation of stem-forming nucleotides is opposite to that of the loop. This is achieved by connecting one stem-forming fragment to the loop via 5′-5′ phosphodiester bonds, while another stem-forming fragment by 3′-3′ bond (Figure 5). Overall, the terminal stem-forming sequences are complementary and can base-pair with each other, but incapable of doing so with the target because of the parallel rather than antiparallel orientation. In another design, stem nucleotides were substituted by optical isomers of natural nucleotides, L-NMPs [101]. L-DNA can only form stable duplexes with complementary L-DNA, but not with D-DNA of the target. The same effect was achieved by using other artificial nucleotide analogs, nucleic acids with bis(hydroxymethyl)benzene-phosphate backbone [102], artificially expanded genetic information system (AEGIS) [103–105], DNA analog that forms adenine-adenine base pairs (Homo-DNA) [106], and potentially can be achieved using x-DNA introduced by Kool and colleagues [107]. Conceivably, the aforementioned strategies allow the design of a series of universal stems that would cover the entire useful temperature range. These stems can be optimized once and uniformly used in the design of MB probes against any sequences. The artificial modifications 3′-3′ and 5′-5′ are now commercially available. Unfortunately, other aforementioned modifications are less affordable by the general user. 

**Figure 6:**
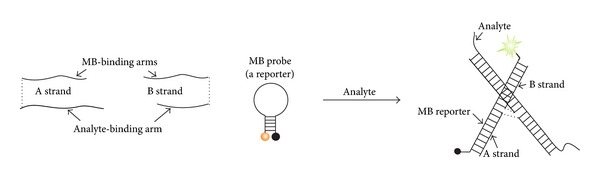
Using a universal MB (UMB) probe as a part of tricomponent X sensor for recognition of specific DNA or RNA sequences [98, 108]. Adaptor *A* and *B* strands hybridize to both analyzed sequence and a universal near-optimal MB probe reporter to form fluorescent tetrapartite complex. Triethylene glycol linkers (TEG) that connect the MB-binding arms with the analyte-binding arms are shown as dashed lines.

An alternative strategy to address the interference issue uses indirect binding of an MB probe to the analyte by the adaptor strands (A and B strands in Figure 6) [98, 108]. Each strand possesses a fragment complementary to the MB probe (MB-binding arms) and another fragment complementary to the target nucleic acid (analyte-binding arms). The MB-binding arms are designed to be short enough to interact only weakly with the MB probe in the absence of the target, thus maintaining low background fluorescence. The presence of a specific analyte leads to hybridization of A and B strands to the target and joining of the two MB-binding arms to cooperatively open the MB reporter. The resultant complex contains MB probe in its opened conformation, thus enabling high fluorescence. The loop interference problem is addressed by designing a near-optimal universal MB (UMB) probe: it contains target-independent A/T-rich loop sequence, which has reduced affinity to nonspecific nucleic acids and short C/G-rich stems, which ensures high hybridization rates along with the high stability. The stem interference problem is re-solved by the absence of the direct MB probe-analyte contacts and by the freedom in the nucleotide choice. We named this probe *X sensor*, since it forms a single DNA crossover (X) structure (also known as DNA four-way junction).

### 2.3. SNP Genotyping

Even before the invention of MB probe, there had been an understanding that a conformational constraint added to a hybridization probe can significantly improve hybridization specificity [30]. However, only with the introduction of the practically significant format of MB probe, this idea has found broader recognition due to the work of Bonnet et al. [29]. 

**Figure 7:**
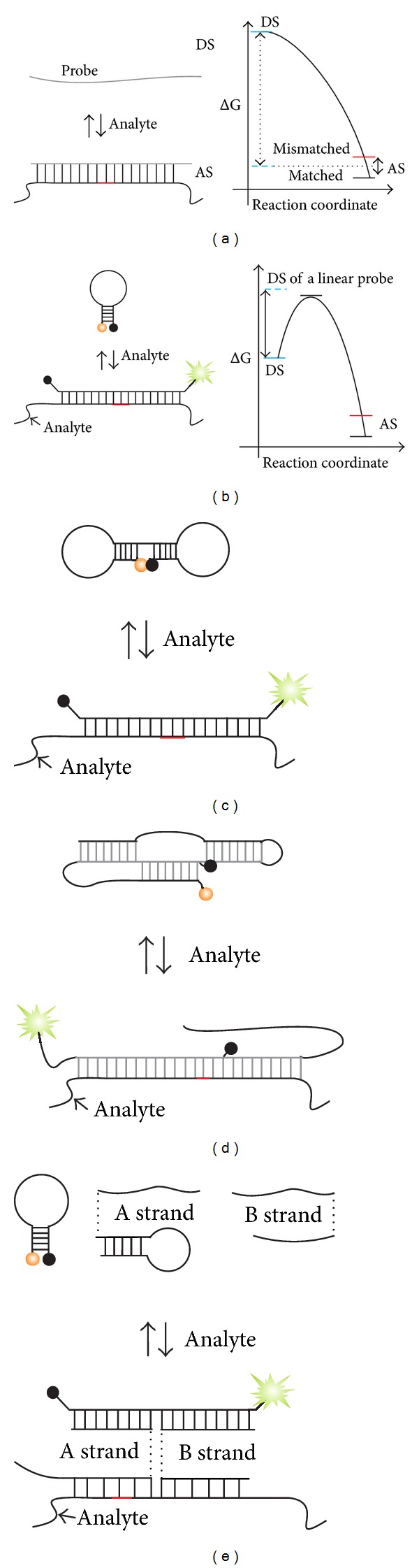
Schemes and energy diagrams for hybridization probes. (a) Hybridization of a linear oligonucleotide probe. “DS” and “AS” stand for probe-analyte-dissociated and -associated states, respectively. The difference in the energy between matched and mismatched duplexes is much smaller than the energy gap between DS and AS. (b) Hybridization of the MB probe. The energy of DS is reduced due to the formation of a 4–7-nucleotide stem. The maximum on the energy curve corresponds to the DS of the MB probe with unwound stem. (c) Dumbbell MB probe contains two stem loop elements in its structure. (d) Triple-stem probe has three stem structures. (e) Hybridization of MB-based X sensor. X sensor of Figure 6 was equipped with an additional stem-loop in the analyte-binding arm of *A* strand. *A* strand binds the analyte within an SNP site.

The higher the affinity of a hybridization probe to a target nucleic acid, the lower its specificity [109]. Indeed, the formation of 15–20 base pairs between a probe and an analyte is required to uniquely bind a particular sequence within a genome. Hybrids of such length are too stable to be sensitive to a single mismatch, since a single mispairing introduces an energetic penalty equivalent to only a small fraction of the total energy gained upon duplex formation (Figure 7(a), right panel). SNP genotyping requires the conditions when the probe-analyte dissociated state (DS) is positioned between the energies of the associated states (AS) for matched and mismatched duplexes (Figure 7(a), right panel, dashed blue line). Under such conditions, the fully matched duplex is formed, while the mismatched one is dissociated. 

MB probe forms a competing secondary structure in DS when unbound to the analyte. The reduction of the free energy of DS is achieved due to Watson-Crick base pairing in the stem part of the hairpin (Figure 7(b), energy diagram). It was demonstrated that MB probes distinguish mismatches over a wider temperature range than unstructured probes do [29]. However, in practice, MB probe-based genotyping requires measuring probe-target melting profiles, which implies using sophisticated equipment, and extends the assay time. MB probes with longer stems have improved mismatch discrimination ability but reduced hybridization rates [86]. 

To further strengthen the conformational constraint and thus improve SNP differentiation ability, more than one stem can be introduced into a hybridization probe. For example, Lv et al. suggested dumbbell-shaped MB (DMB) probe [120] (Figure 7(c)). The probe's terminal fragments are complementary to the internal positions of the target-specific fragment. This design allowed an improved SNP recognition at 20 and 37°C with the discrimination factor ((*F*_matched_ − *F*_*o*_)/(*F*_mismatched_ − *F*_*o*_)) of up to 60. A possible advantage of DMB design over conventional MB probe might be the close dislocation of a fluorophore and a quencher, which potentially may result in an even more efficient quenching. DMB design may appear to produce efficient quenching if it uses nucleotides with a fluorophore and a quencher directly attached to deoxyribose, such as developed earlier by Asanuma [110, 111] and colleagues for in-stem MB probes (see Figure 9(b) below). The hybridization kinetics of DMB probe, however, was slower than that of the traditional MB probe. Indeed, the high DMB stability provided a kinetic barrier for the rearrangement required upon formation of the probe-target duplex. Additional disadvantage is the dependence of the stem sequences and, therefore, their stabilities on the sequence of targeted analytes. This limitation is absent in the traditional MB design but may narrow the scope of possible analytes for DMB probe. 

Xiao et al. developed “triple-stem DNA probe” (Figure 7(d)) with even greater-level of conformational constraint [121]. The probe is a single-stranded oligodeoxyribonucleotide that is folded in a compact secondary structure with three separate relatively short (7 nucleotides) stems. It hybridizes to a target analyte by consecutively unwinding the three short stems, which is more kinetically favorable than unwinding of one long stem. In this stepwise process, the high activation-energy barrier is divided into three lower barriers [31]. High probe selectivity toward mismatched analyte was maintained over a wide temperature range of 20 to 60°C. The hybridization rate, however, was lower (signal saturation occurred after 3 hrs) than that of MB probe (typical hybridization time is 5–15 min).

We found that an MB-based tricomponent X sensor (Figure 6) allows very high level of selectivity at ambient temperatures [98, 108]. In this case, the decrease in the free energy of the probe's unbound state is likely to be achieved due to the entropy factor, since the probe dissociates into three fragments allowing significant entropy gain. Furthermore, the design of the X sensor allows introduction of one (Figure 7(d)) or two stem loops [108] in addition to the stem loop of MB probe. As a result of such design, the sensor contains up to three stems, which represent a very high level of conformational constraint. Importantly, hybridization of X sensor was almost completed in 15 min [122], which is comparable with the hybridization of conventional MB probes. The probe detected a matched analyte in the presence of 100 times excess of a single-base mismatched target at room temperature [108]. These performances of X sensor might be useful for the detection of specific RNA or DNA sequences in complex mixtures under near physiological conditions.

### 2.4. Analysis of Folded Nucleic Acids

MB probe design does not favor the detection of RNA or single-stranded DNA folded in stable secondary and tertiary structures. Indeed, the hairpin-shaped structure of MB probe hinders hybridization with the targeted sequence. The presence of a conformational constraint in a target disfavors hybridization even more. MB probe was shown to hybridize inefficiently to stem-loop-folded RNAs or DNA [123–125]. On the other hand, the majority of naturally occurring RNA sequences are folded in stable secondary and tertiary structures. A common approach to address this problem is to target single-stranded fragments of the analyzed nucleic acids. This approach, however, severely limits the choice of the targeted sequences within naturally occurring nucleic acids. Moreover, in cells RNA might be bound by proteins, which further limits accessibility of a target sequence. For example, Rhee et al. attempted to design MB probes for detection of a specific mRNA in live human dermal fibroblasts cells [126]. Out of ten MB probes tested, only two were efficient in reporting mRNA in cells. Importantly, some MB probes selected based on recommendations of RNA folding software did not work well. Therefore, there is a need for a robust fluorescent sensor that allows detection of RNAs folded even in the most stable secondary structures.

**Figure 8:**
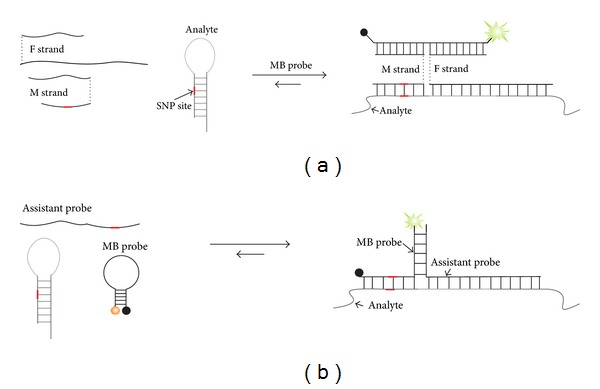
Analysis of structured nucleic acids using the helper DNA strands. (a) MB-based tricomponent sensor. F strand uses long analyte-binding arm to unwind the analyte's secondary structure. M strand binds to the opened analyte fragment only if an SNP site matches the analyte-binding arm of M strand. The two adaptor strands joint by the analyte open MB probe, thus producing high fluorescence. (b) Y-shaped junction probes. An assistant probe tightly binds and unwinds the analyte and together with MB probe forms fluorescent three-way junction complex. MB probe is partially complementary to the SNP site.

Recently, we adopted the X sensor for the analysis of nucleic acids folded even in very stable structures [122, 124, 125]. In this approach, the X sensor (Figures 6 and 7) was equipped with an adaptor strand F containing a long analyte-binding arm (Figure 8(a)). This arm tightly bound the analyte and unwound its structure to enable the hybridization of the second adaptor strand and, finally, the formation of a signal-reporting complex. Importantly, the assay was carried out at ambient temperatures to unwind a stem-loop-folded DNA with the *T*_*m*_ > 80°C [109]. Notably, allele discrimination was achieved even for the SNP site located in the stem-forming analyte fragments. This approach allows detection of subnanomolar concentrations of folded RNA at ambient temperature with excellent selectivity [122]. 

An approach that uses one helper oligonucleotide along with an MB probe to open a folded analyte was suggested recently by Li et al. [127] (Figure 8(b)). The approach was shown to have excellent SNP differentiation ability. In contrast to the X sensor, this approach uses MB probe with analyte-dependent sequences, which requires synthesis of a new MB probe for each new analyte.

### 2.5. Improving Signal-to-Noise (S/N) Ratio

Signal increase upon hybridization to the target is the fundamental phenomenon that has made possible the success of MB probe. The higher the fluorescence increase upon MB probe hybridization to a target (S/N ratio), the lower limit of detection (LOD) and the broader the dynamic range. S/N ratio can be increased in two ways. First, the fluorescent intensity of the fluorophore can be improved; second, the background can be reduced.  Table 1 summarizes available data for combinations of fluorophore/quencher pairs to illustrate a possible range of S/N and LOD for the variations of MB probes. 

**Figure 9:**
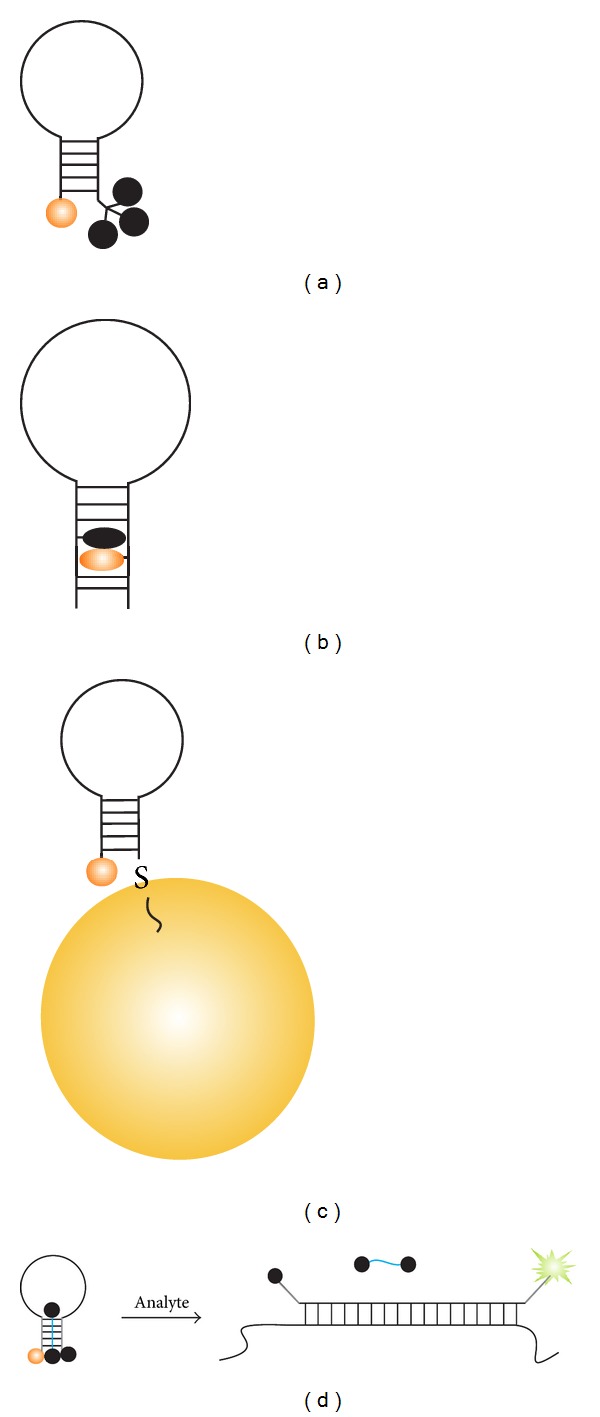
Strategies to reduce the background fluorescence of MB probes. (a) Superquenched MB probe contains more than one quencher [75]. (b) In-stem probe takes advantage of stacking interaction of stem base pairs with a fluorophore and a quencher dyes to achieve efficient contact quenching [110, 111]. (c) Gold nanoparticle-conjugated MB probe takes advantage of superior quenching abilities of gold nanoparticles [74]. (d) Triplex-forming MB probe makes use of more than one quencher [112] to reduce the background fluorescence.

The design of MB probe provides a perfect platform for reducing the background fluorescence by bringing a fluorophore and a quencher in close proximity [28, 128]. To ensure efficient quenching, the fluorophore/quencher pair should be chosen based on recommendation by Marras [28]. Other strategies to improve quenching efficiency and lower the background fluorescence are shown in Figure 9.

Yang et al. suggested using multiple quencher dyes to improve quenching efficiency (Figure 9(a)) [75]. An S/N ratio of up to 320 was achieved by this approach (Table 1, row 4). Kashida and colleagues developed in-stem MB probe (Figure 9(b)), in which fluorophore/quencher pairs were incorporated into the stem region [110, 111]. Very close location of the fuorophore and the quencher enabled efficient reduction of the MB probe fluorescence in the closed conformation. An S/N ratio of up to 58 was reported with the MB probe containing two perylene/anthraquinone fluorophore/quencher in-stem pairs [111] or up to 70 with Cy3/4′-(dimethylamino)-2-nitroazobenzene pair [110]. Häner et al. developed excimer-controlled MB probe [129, 130], in which the fluorescence of two pyrene residues attached to the same side of the stem was quenched in the closed conformation by two quencher residues located on the opposite side of the stem. The two adjacent pyrenes, however, formed an excimer fluorescent signal in the opened conformation. An S/N ratio of 434 was reported with this approach. This is among the highest S/N ratios (see Table 1). The detection limit of 0.3 nM was achieved [129].

Gold nanoparticles were shown to efficiently quench the fluorescence of various common dyes to rise S/N ratio of MB probe up to 100 (Figure 9(c)) [74]. An original approach is *triplex* peptide nucleic acid (PNA) MB probes suggested by Grossmann et al. [112]. In this design, the 5′- and 3′-termini of the probe were supplemented with T_8_ or T_11_ PNA sequences. In the presence of an oligo A PNA, the stem fragments of the probe formed a triplex structure (Figure 9(d)). Binding to a target led to dissociation of the triplex-forming helper PNA. When the PNA helper was supplemented with an additional quencher, the quenching efficiency was improved.

Increasing the absolute fluorescence of MB probe in the extended conformation is an important task. For example, monitoring nucleic acids in cells requires intensive light emission to produce a signal above the natural fluorescent background of live cells. To achieve an intensive fluorescence, Yang et al. [76] conjugated an MB probe with a fluorescent polymer poly(phenylene ethynylene). The polymeric fluorophore was shown to generate much brighter fluorescence than regular organic fluorophores. A very impressive LOD for an MB probe of 1 pM was reported by Krasnoperov et al. for MB probe labeled with a lanthanide-based luminescent complex [79]. This detection limit is about 1000 times lower than that of a regular MB probe and at least 30 times lower than that of other lanthanide-based fluorescent techniques [131]. Note that the lowest detection limit correlates with one of the highest S/N ratios (Table 1). This outstanding probe performance was attributed to both the sharp and intensive luminescence of the lanthanide complex and efficient quenching of the lanthanide luminescence in the closed conformation of the probe [79].

Improving S/N ratio of MB probes has been the subject of multiple investigations in the last decade, while most applications have been done with the conventional simple and affordable design. Indeed, increasing S/N ratio is less important for the most practically significant application of MB probe, rtPCR, since PCR itself provides high degree of DNA amplification, and thus, the assay sensitivity is not limited by the probe performance. Lowering the detection limit, however, is extremely important for dynamic monitoring of nucleic acids in cells. 

### 2.6. Synthetic Cost

Due to its high selectivity, the MB probe is an attractive tool for genotyping single-nucleotide polymorphisms (SNPs). Genotyping of each SNP site requires synthesis of at least two MB probes, each of which is specific to one allele. Moreover, MB probe optimization often includes testing of several alternative designs for the same target. Currently custom-synthesized MB probes are available from a number of vendors for the price of $400–850 per a probe [88, 89]. This price is greater than that of TaqMan probes and cannot be afforded by low-budget laboratories, especially if multiple SNP sites are to be analyzed. Several factors contribute to the high cost. First, synthesis of MB probe requires conjugation with two organic dyes, which is relatively a rare modification and thus cannot be introduced cost-efficiently during the automated DNA synthesis. Second, the probe's preparation requires postsynthetic HPLC purification to remove possible fluorescent impurities which, if not removed, create high background. The third factor is high royalties. This high price of MB probes has been a driving factor for the development of alternative technologies including label-free electrochemical detection of nucleic acids [132–134]. 

**Figure 10:**
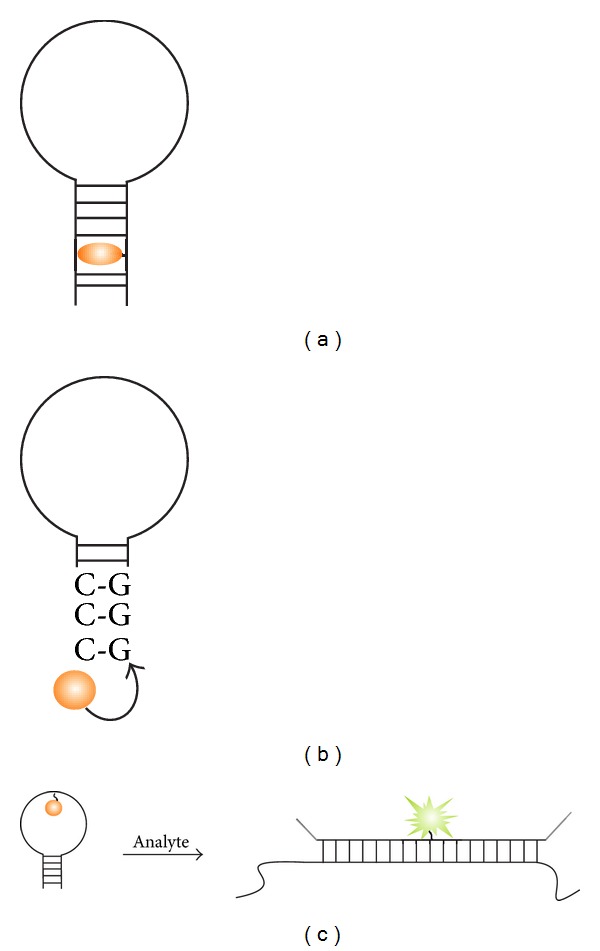
A variety of quencher-free MB probes. (a) Quencher-free in-stem MB probe developed by Kashida et al. [113]. (b) “Smart” probes take advantage of multiple guanine residues located in the stem to quench the fluorescence of a fluorophore dye [114]. (c) Base-discriminating fluorescent (BDF) probe. The fluorescence of a loop-conjugated fluorophore is quenched by the loop nucleotides [115]. Hybridization of the probe to the cognate analyte leads to dequenching and increasing the probe's fluorescence.

Some cost reduction can be achieved using “quencher-free” MB probes [61], some of which avoid conjugation of a fluorophore-labeled hairpin oligonucleotide with a quencher dye. Variations of such MB probes are shown in Figure 10. For example, Kashida et al. described quencher-free MB probe containing 7-hydroxycoumarin as a fluorescent reporter [113] (Figure 10(a)). Interestingly, the pKa of in-stem bound dye was 1.2 units higher than that in a single-stranded DNA. Up to tenfold fluorescent increase can be achieved upon the target hybridization to the probe in a buffer with pH 8.0. Heinlein et al. introduced a “smart” probe, in which fluorescence of a 5′-conjugated fluorophore was quenched by the guanine residues [114] (Figure 10(b)). Fluorescent increase of up to 20-fold can be achieved with this approach. Okamoto et al. introduced base-discriminating fluorescent (BDF) probe [115]. In BDF probe, pyrene group was covalently conjugated to a loop nucleotide (Figure 10(c)). The surrounding nucleotides quenched the pyrene fluorescence in the unbound form. However, when probe was hybridized to the complementary target, the nucleotides formed Watson-Crick base pairs, which reduced the quenching effect. An S/N ratio of up to 8 was achieved with such probes.

A major cost reduction for an MB-based assay can be attained using indirect binding of MB probe to target analytes (Figure 6). In this case, a single optimized UMB probe can be used for the analysis of a great variety of targets. There are two stages of cost saving with this approach. Primarily, the approach eliminates optimization of the fluorescent reporter for each new analyte. With the X sensor of Figure 6, the fine-tuning of the sensor's performance requires changing only relatively inexpensive dye-free adaptor strands, which is more affordable than screening a series of MB probes required by the classical optimization scheme. Secondary, UMB probe can be synthesized in bulk amounts and used efficiently without leftover in multiple applications. 

**Figure 11:**
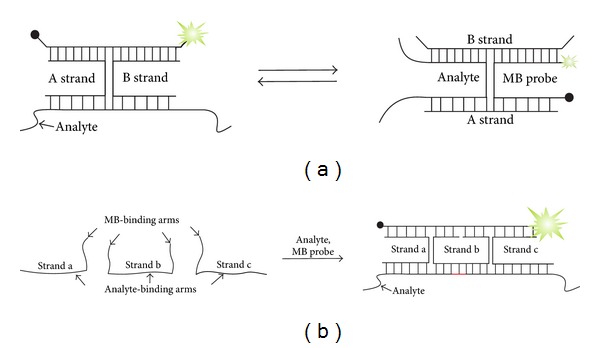
DX tile-based sensor for cost-efficient detection of multiple analytes. (a) Two conformations of the MB probe-containing DNA four-way junction. (b) Principal scheme for DX tile-based sensor. Adaptor strands a, b, and c hybridize to an MB probe and an analyte to form a fluorescent complex containing two DNA four-way junctions.

Additional cost improvement for multicomponent MB-based sensors is possible with DX tile-based multicomponent sensor [135]. An important structural feature of the MB-based X sensor (Figure 6) is the triethylene glycol linkers (TEG) that connect the MB-binding arms with the analyte-binding arms of the adaptor strands (Figure 6). TEG linkers fix one of the two possible conformations of the DNA four-way junction complex, in which the fluorescence of MB reporter is the highest (Figure 11(a)). Using the TEG-modified adaptor strands, it is possible to reduce the cost of multiplex assays by ~5 times in comparison with the conventional MB approaches. Replacement of all adaptor strands with unmodified DNAs would additionally reduce the reagent cost by about 4-5 times, making the total cost saving ~23–34 times. This was achieved by adopting Seeman's DX tiles [136] to design DX tile-based multicomponent sensor [135]. The DX motif-forming sensor takes advantage of the three adaptor strands a, b, and c, which cooperatively hybridize to both the analyzed nucleic acid and the MB reporter and form the double crossover antiparallel structure (Figure 11(b)). In the resultant DX motif-containing complex, the MB probe is fixed in the elongated conformation, providing the high fluorescent output. 

Overall, the traditional MB probe remains to be an expensive tool for instantaneous SNP-specific nucleic acid analysis. Using multicomponent sensors in conjunction with the optimized universal MB probe may significantly reduce the cost of multiple assays and make it affordable even for the low-budget laboratories.

## 3. Recent Variations of MB Probes

This section highlights some of the most recent and exciting developments in the field of MB probes and related instantaneous probes. Wang et al. [137] reported caged MB probe, in which stem structure was stabilized either by a covalent bond between the fluorophore and the quencher or by means of biotin-streptavidin interactions. In the caged form, MB probe produced only low fluorescent signal even in the presence of an analyte. A pulse of light, however, cleaved a photosensitive linker, thus forcing the MB probe to report the presence of the analyte. Another example of a light-activatable MB probe was reported by Joshi et al. [138]. In their design, the loop sequence was equipped with caged nucleotides that could not form Watson-Crick base pairs. The probe was activated by light irradiation (366 or 405 nm) to remove caging and enabling hybridization to the target. Caged MB probes might be useful for speciotemporal monitoring of RNA in live cells. 

**Figure 12:**
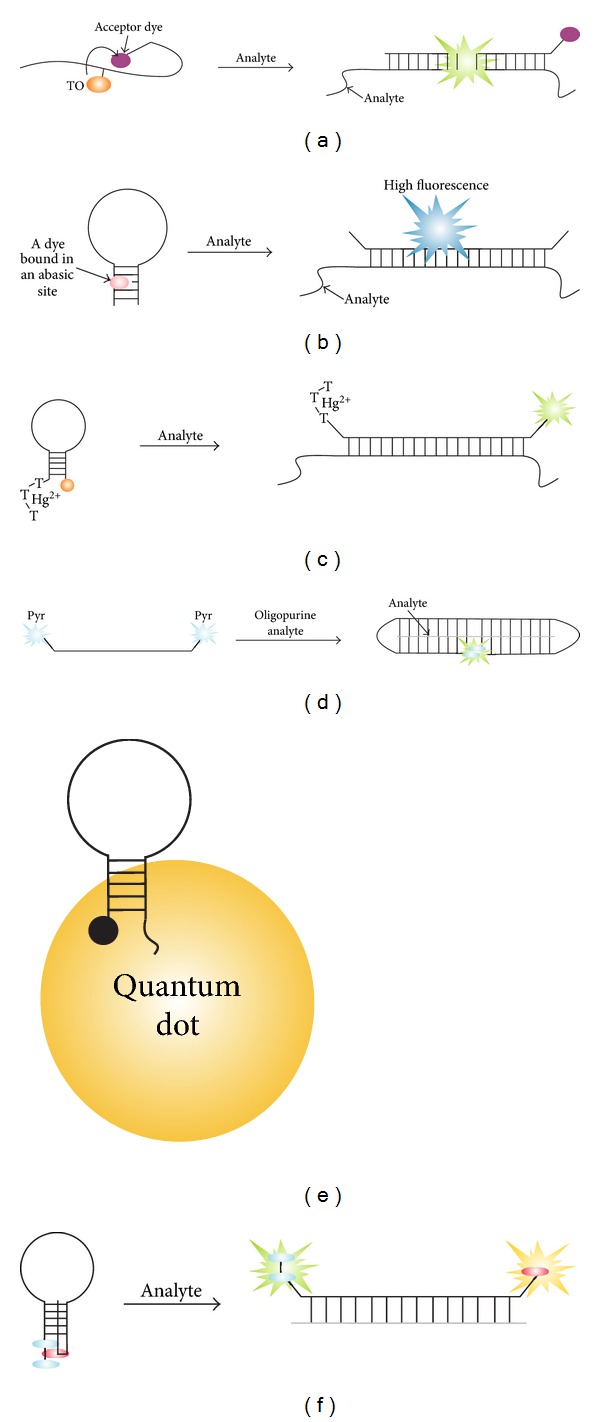
Recent variations of MB probes. (a) Dual fluorophore PNA FIT probes [116]. The flexibility of a single-stranded PNA chain enables the contact of thiazole orange dye (TO) with the acceptor dye (fluorescent or dark quencher), which results in efficient quenching of TO fluorescence. The fluorescence increase upon target binding is achieved due to (i) separation of the fluorescent donor from the fluorescent acceptor and (ii) increase in fluorescence as a result of TO stacking interactions with newly formed base pairs. (b) Label-free MB probe (APMB) [117]. When noncovalently bound to an abasic site within the stem structure of the probe, a dye produces low fluorescent signal. Hybridization to a complementary target releases the dye free in solution, thus enabling high fluorescence. (c) Thymidine-terminated MB probe [118]. The fluorescence of FAM group in the closed conformation of the probe was quenched due to the binding of Hg^2+^ ions by three consecutive thymidine residues on the opposite terminus of the probe. (d) Triplex-forming DNA probes [116]. (e) Quantum dot-(QD-) based MB probe [117]. (f) Two-color MB probe [119]. Cyan ovals are two pyrene residues, which interact with perylenediimide residue (red). Hybridization of an analyte separates pyrene dimer form perylenediimide, thus increasing the fluorescence of both dyes.

Socher et al. designed a new variation of stemless MB probe, named FIT probe (Figure 12(a)) [116]. The probe was equipped with thiazole orange dye in the internal position and an acceptor dye (fluorescent or dark quencher) attached to one of the probe's termini. In the absence of the target, a flexible single-stranded PNA chain brought TO in contact with the acceptor dye, enabling the fluorescence quenching. Upon the target binding, the fluorescence was restored, since TO was separated from the quencher. Additional increase in fluorescence was achieved since TO was involved in stacking interactions with newly formed base pairs. Impressively, an S/N of the probe was 450, which resulted in the detection limit of 40 pM. This PNA rather than DNA-based probe is promising for the detection of RNA targets in biological samples and live cells. Sato et al. [117] reported a label-free MB probe that takes advantage of abasic site introduced in the MB stem (Figure 12(b)). Fluorescent dyes 2-amino-5,6,7-trimethyl-1,8-naphthyridine (ATMND) or lumiflavin reduced their fluorescence when bound to a DNA abasic site. However, the dye regained high fluorescence when the stem loop was disrupted due to the hybridization to a target analyte. In the presence of the analyte, a 17-fold fluorescence increase was achieved. This design should be considered as cost-efficient in comparison with a conventional MB probe as it does not require covalent conjugation of dyes with an oligonucleotide. 

Hou et al. reported a variation of quencher-free MB probe, in which three dangling thymidine residues were conjugated to the 5′ end, while FAM fluorescent group was attached to the 3′ end (Figure 12(c)) [118]. The thymidines were able to bind mercury (II) from solution to form an efficient quencher. The probe, however, was able to produce an S/N ratio of only ~40%. Asanuma et al. improved the in-stem MB probe by incorporating three fluorophore/quencher pairs in stem portion of an MB probe [139]. In the presence of poly(L-lysine)-graft-dextran, an impressive S/N ratio of 570 was achieved. This is one of the highest S/N ratios reported for MB probes (see Table 1). Yi et al. [140] improved earlier reported MB probe that uses graphene oxide as a quencher [141, 142]. An S/N ratio of 31 was achieved [140].

Rosa et al. used gold nanoparticle-quenched MB probe (similar to that shown in Figure 9(b)) for transcription monitoring [143]. The sensor was used to measure the rate of *in vitro* RNA synthesis. Interestingly, DNA-based probes were used in this study despite the previous report that showed that stem-loop-folded MB probes can be opened by nonspecifically synthesized RNA products under the *in vitro* transcription conditions [144]. A possible explanation for such beneficial behavior is that the conjugation with gold nanoparticle might prevent the nonspecific recognition of the DNA hairpin structure by T7 RNA polymerases. Dunams et al. used nuclease-resistant 2′-O-methyl thiolate MB probe to detect replication of adenovirus and echovirus in cells using fluorescence microscopy [119]. Thus, MB probe provided a tool for instantaneous quantification of viral infectivity.

Sato et al. reported a new variation of in-stem MB probe (Figure 9(b)) in which both perylene fluorophore and anthraquinone quencher were attached to the 5th position of thymine residues [145]. An S/N ratio of 24–50 was achieved with such probes. Li et al. suggested using DNA triplex formation between a probe and a target [146], to produce a signal from a pyrene excimer. The probe was conjugated with two pyrene residues at its opposite termini, which is similar to that of other excimer-forming MB probe (Table 1, row 10). The linear probe was able to bind oligopurine DNA sequences by forming a DNA triplex structure (Figure 12(d)). In this complex, the two pyrene residues formed excimer, which could be detected by the red shift in fluorescence. The probe was found to be sensitive toward single-base substitution in the analyte in a broad temperature range.

Wu et al. used quantum dot- (QD-) based MB probe for *in situ* detection of *E. coli β*-lactamase gene [147]. In this variation, QDs were used as a fluorophore (Figure 12(e)) [141]. The advantage of such replacement is greater resistance of QD to photobleaching as well as improved cellular uptake. However, a fluorescence increase of only fourfold was produced in the presence of the target. Yin et al. used QD as a fluorophore and gold nanoparticle as a quencher in the MB probe design [148]. An S/N ratio of 7.3 was achieved. This type of MB probe was applied for the study of virus replication in living cells. 

Biner and Häner developed a two-color MB probe [149]. In this probe, two pyrene derivatives formed a sandwich associate with a single perylenediimide residue on the edge of a triple helix stem (Figure 12(f)). The sandwich arrangement enabled efficient quenching of the fluorescence of both dyes. In open conformation, however, both dyes produced characteristic signal at two different wavelengths. An advantage of the two-color detection is the reduced chance of false-positive signal [130, 149].

Kang et al. simultaneously detected two microRNAs in live cells with the help of the two MB probes equipped with FAM or Texas Red-X fluorophores [150]. Even though PNA-based MB probes have been already used for microRNA imaging in the past [151, 152], this is the first example of using multiplexing capabilities of MB probes for the detection of microRNAs. Another interesting application of MB probe approach was reported by Guetschow et al. [153]. The authors employed the probe to detect an RNA biomarker for breast cancer metastasis. The detection limit was found to be 167 nM, and the biomarker was detected directly in serum samples.

## 4. Conclusion

MB probes have been successfully applied as commercial products in rtPCR format. Another promising application of MB probes is the analysis of intracellular RNA molecules. This application is currently under extensive development. To ensure high reliability of the results, MB probes need to be optimized for each task. Importantly, the strategy for the MB probe optimization depends on the chosen application. For example, for intracellular use in nucleic acid analysis, such parameters as high nuclease stability and high S/N ratio are important, while for application in rtPCR format, the most important characteristics are capability of multiplexing and accurate allele discrimination. New applications of MB probes include the detection of microRNA [150–152, 154, 155] and small molecules [156, 157], as well as employing the probe in DNA nanotechnology [158–162].

## Figures and Tables

**Table 1:** Variations of 5′-X- and 3′-Y-terminal attachments to increase signal-to-noise (S/N) ratio of an MB probe.*

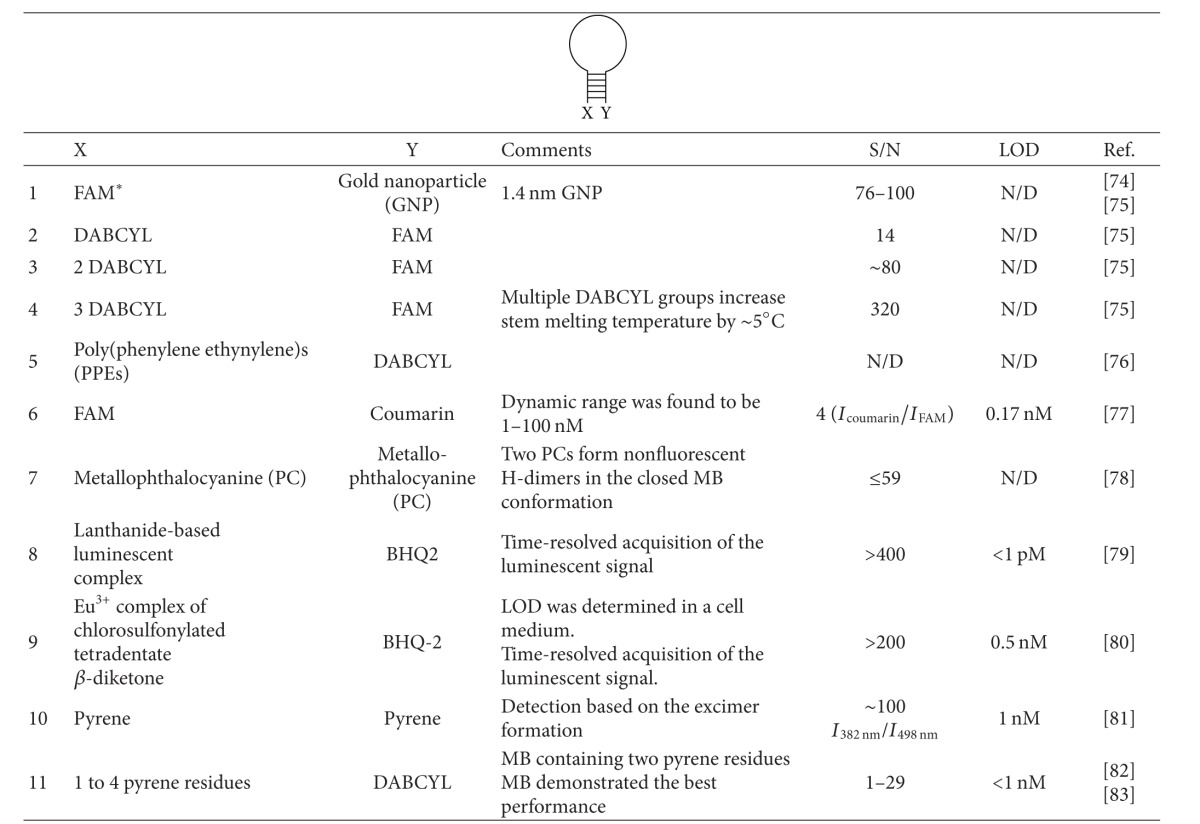

*N/D: not determined; FAM: fluorescein; DABCYL: 4-((4-(dimethylamino)phenyl)azo)benzoic acid; BHQ2: blackhole quencher 2; LOD: limit of detection; S/N: signal-to-noise ratio.
